# Temporal Regulation of Cytokines by the Circadian Clock

**DOI:** 10.1155/2014/614529

**Published:** 2014-04-06

**Authors:** Atsuhito Nakao

**Affiliations:** ^1^Department of Immunology, University of Yamanashi, Faculty of Medicine, 1110 Shimokato, Chuo, Yamanashi 409-3898, Japan; ^2^Atopy Research Center, Juntendo University School of Medicine, 2-1-1 Hongo, Bunkyo-ku, Tokyo 113-8421, Japan

## Abstract

Several parameters of the immune system exhibit oscillations with a period of approximately 24 hours that refers to “circadian rhythms.” Such daily variations in host immune system status might evolve to maximize immune reactions at times when encounters with pathogens are most likely to occur. However, the mechanisms behind circadian immunity have not been fully understood. Recent studies reveal that the internal time keeping system “circadian clock” plays a key role in driving the daily rhythms evident in the immune system. Importantly, several studies unveil molecular mechanisms of how certain clock proteins (e.g., BMAL1 and CLOCK) temporally regulate expression of cytokines. Since cytokines are crucial mediators for shaping immune responses, this review mainly summarizes the new knowledge that highlights an emerging role of the circadian clock as a novel regulator of cytokines. A greater understanding of circadian regulation of cytokines will be important to exploit new strategies to protect host against infection by efficient cytokine induction or to treat autoimmunity and allergy by ameliorating excessive activity of cytokines.

## 1. Introduction

Several parameters of the immune system exhibit “circadian rhythms” which refer to daily oscillations with a period of approximately 24 hours [1, 2]. For instance, the number of specific immune cells (e.g., monocytes, neutrophils, and lymphocytes) in circulation and plasma levels of cytokines (e.g., TNF-*α* and IL-6) show ~24-hour daily rhythm that is translated into variations in acute response to infection [1, 2]. However, it remains poorly understood how such circadian immunity occurs. Recent studies reveal that the internal time-keeping system “circadian clock” is responsible for driving the circadian rhythms evident in the immune system. This review briefly summarizes new studies that provide mechanistic insights into how the immune system is under tight control of the circadian clock. In particular, this review puts emphasis on temporal regulation of cytokines by the circadian clock because cytokines play a central role in shaping immune response as crucial mediators for intercellular communication among immune and nonimmune cells. Better understanding of an emerging role of the circadian clock as a novel regulator of cytokines will be greatly helpful to improve timing of conventional treatment and to exploit new drug targets for immune-related diseases such as infection, autoimmunity, and allergy.

## 2. What Is the Circadian Clock?

The circadian clock drives the daily rhythms in behavior and physiology (e.g., sleep-wake cycles, body temperature, blood pressure, and hormonal secretions) that enable organisms to keep track of the time of day according to daily changes in light intensity [3, 4]. Mammalian circadian clock consists of the central oscillator located in the suprachiasmatic nucleus (SCN) of the hypothalamus and peripheral oscillators virtually present in all cell types [3, 4]. Light activates a specific group of photoreceptors in the retina connected to the central SCN clock which entrains peripheral circadian clock via neural and endocrine pathways. The molecular mechanisms of rhythm generation are cell autonomous, highly conserved in the SCN and peripheral cells, and created and maintained by transcriptional-translational feedback loops that consisted of several “clock genes” and their protein products. Briefly, two transcription factors, CLOCK and BMAL1, activate the transcription of the* Period *(*Per*) and* Cryptochrome* (*Cry*) genes. The* Per* and* Cry* proteins in turn inhibit their own expression by repressing CLOCK/BMAL1 activity. This negative feedback loop, with additional posttranscriptional modifications, generates ~24-hour oscillations of the clock protein levels and activity, which are translated into periodic changes of a variety of clock-controlled genes (CCGs) involved in important biological processes, including immune responses.

## 3. Circadian Regulation of Immunity 

It has been well documented that many parameters in the immune system exhibit circadian rhythms [1, 2]. These include the number of circulating hematopoietic cells and the levels of cytokines. Not only the components of the immune system but also the immune functions, such as leukocyte migration/trafficking, and innate immune response also show circadian rhythms [1, 2]. However, biological basis behind those observations has been unclear. Recent excellent studies have provided compelling molecular evidence that the circadian clock is responsible for the temporal activities in the immune system [5–8].

Scheiermann et al. have recently reported that the central SCN clock drives circadian rhythms in the expression of adhesion molecules (e.g., ICAM-1 and VCAM-1) on endothelial cells or chemokines/chemokine receptors (e.g., CCL2 and CXCR4) in tissue or leukocytes, which contributes to a time of day-dependent recruitment of leukocytes into the tissues such as the bone marrow and muscle [9]. The circadian activity of sympathetic nervous system driven by the central SCN clock likely regulates the expression of adhesion molecules or chemokine/chemokine receptors in a temporal manner, thereby controlling leukocyte migration.

Nguyen et al. suggest another regulatory mechanism for leukocyte migration by the circadian clock [10]. They found that frequency of Ly6C^high^ inflammatory monocytes (an innate immune cell type for the first line of defense against pathogens) in blood, spleen, and bone marrow exhibited circadian oscillations, which corresponded to the diurnal variations in recruitment of the cells into the sites of inflammation. They suggested that CLOCK/BMAL1 heterodimer negatively regulated expression of CCL2 in monocytes, which likely contributed to the circadian oscillations of Ly6C^high^ inflammatory monocytes. In contrast to the study by Scheiermann et al. [9], this study therefore puts more emphasis on an important role of peripheral immune cell clock in leukocyte migration.

Silver et al. have provided molecular evidence how the circadian clock controls innate immune response [11]. They found that CLOCK/BMAL1 heterodimer bound to the promoter of Toll-like receptor (*Tlr9*) and promoted the expression and function in a circadian manner. Consistent with the findings, they showed that mice immunized at the time of enhanced TLR9 responsiveness presented strong innate immune reactions with an improved adaptive immune response.

Most recently, T cell development has been surprisingly shown to be regulated by the circadian clock proteins. Yu et al. reported that CLOCK/BMAL1-mediatd REV-ERB*α* induction inhibited expression of NF-IL3, a suppressor of Th17 cell development, thereby selectively influencing Th17 cell development [12]. Interestingly, circadian disruption by chronic light-cycle perturbations increased Th17 frequency in mouse intestine and worsened dextran-sodium sulfate (DSS)-induced colitis in mice [12]. Several other important immune functions have been also shown to be regulated by the circadian clock and the reader is encouraged to consult with excellent reviews for detailed overview of circadian immunity in many aspects [5–8].

## 4. Circadian Clock Proteins Temporally Gate Inflammatory Cytokine Expression

Cytokines are essential to shape immune responses and are indispensable for self-defense against infection. On the other hand, once the cytokine network goes out of control and an excessive amount of cytokines are produced, serious damage, such as sepsis or autoimmunity and allergy, occurs in the host animals. Therefore, expression of cytokines should be tightly regulated to maintain homeostasis of the immune system and, indeed, cytokine expression is regulated at multiple levels. Recent excellent studies reveal that the circadian clock is also an important regulator of cytokines [5–8]. Because circadian regulation of cytokines is extensively studied in* in vitro* and* in vivo* models of endotoxin LPS-induced inflammatory responses in innate immune cells, this review summarizes such studies to describe how the circadian clock regulates cytokine expression at the molecular levels.

Keller et al. reported that mouse macrophages derived from spleen, lymph nodes, and peritoneal cavity contain intrinsic circadian clockworks that operate autonomously as well as other innate and acquired immune cells [13]. Macrophages isolated from mouse spleen stimulated with LPS at different time points display circadian rhythms in TNF-*α* and IL-6 secretion and the cytokine secretion rhythms persist in constant* in vitro* culture conditions, suggesting that macrophage-intrinsic circadian clock may govern these oscillations [13]. Interestingly, ~8% of the expressed genes in peritoneal macrophages show circadian modulation including the genes involved in LPS-immune response pathways such as MD-1 and CD180 [13], which may explain rhythmic secretion of the cytokines upon LPS challenge.

It has been known since pioneering work of the 1960s and 1970s that mice challenged with LPS showed significant circadian-dependent variation in magnitude of response including mortality and cytokine concentrations in serum. Gibbs et al. showed that the temporal variations in serum IL-6 following LPS challenge were absent in mice with specific deletion of BMAL1 in myeloid cells [14], suggesting that myeloid cell-intrinsic clockwork provides temporal gating of cytokine responses to LPS. BMAL1 activates transcription of the nuclear receptor REV-ERB*α* and REV-ERB*α*, which in turn inhibits BMAL1 transcription, forming an accessory feedback loop that stabilizes the circadian clock machinery [4]. Because peritoneal macrophages exhibit a profound temporal variation in transcriptional factor REV-ERB*α* (~20-fold differences across the day) and the temporal variation of REV-ERB*α* was completely suppressed in Bmal1-deficient peritoneal macrophages, Gibbs et al. investigated whether REV-ERB*α* was a key molecule linking the circadian and inflammatory pathways. As expected, rhythmic immune responses to LPS were abolished in REV-ERB*α*-deficient mice, suggesting a link among BMAL1, REV-ERB*α*, and IL-6 production in macrophages upon LPS challenge [14]. The precise biochemical mechanisms remain to be determined.

Spengler et al. showed that the severity of LPS-induced inflammatory response in mice was correlated with the intensity of NF-*κ*B activation and that NF-*κ*B activation is, in fact, under circadian control based on experiments using NF-*κ*B reporter mice [15]. They demonstrated that the core circadian protein clock bound to the p65 subunit of NF-*κ*B, acetylated p65, and upregulated NF-*κ*B-dependent transcriptional responses. Interestingly, clock acts as a positive regulator of NF-*κ*B-responsive genes distinct from the transactivation of circadian genes via E-box elements that i, independent of its circadian function [15].

Narasimamurthy et al. reported another mechanism of how the circadian clock controls inflammatory cytokine expression [16]. They showed that* Cry*-deficient macrophages exhibited a marked increase in TNF-*α* and IL-6 protein secretions compared with wild-type macrophages, suggesting that* Cry*-deficient macrophages were hypersensitive to LPS stimulation. They also showed that* Cry* binds to adenyl cyclase, resulting in enhanced levels of cAMP and constitutive PKA activity and NF-*κ*B activity in macrophages [16]. Because* Cry*-deficient macrophages constitutively upregulated TNF-*α* and IL-6 expression, an important implication of this study is that an arrhythmic clock system may be sufficient to increase the stress levels of cells leading to constant expression of inflammatory cytokines and causing a low-grade chronic inflammatory status.

Collectively, these findings provide molecular insights into how the circadian clock regulates daily variations in the induction of inflammatory cytokines upon a powerful challenge of LPS and how disruption of the clockwork affects the cytokine responses. In particular, these findings clearly demonstrate that certain clock proteins interact with important regulatory pathways for cytokine expression. The results may explain the daily fluctuations in susceptibility to various pathogens to the immune system.

## 5. Circadian Regulation of Cytokines Involved in Inflammatory Diseases

Many chronic inflammatory diseases show circadian rhythms in disease severity or symptoms [8]. However, it remains largely unclear whether the circadian clock is attributed to such circadian pathophysiology. Rheumatoid arthritis (RA) is one of the representative chronic inflammatory diseases with circadian pathophysiology. Patients with RA exhibit bad joint inflammation, pain, and stiffness in the morning hours, which is associated with increased inflammatory cytokine production. Recently, Hashiramoto et al. showed the link between RA and the circadian clock for the first time. They demonstrated that* Cry*-deficient mice exhibited enhanced arthritis associated with upregulation of inflammatory cytokines including TNF-*α* [17]. Their data are largely consistent with the findings that* Cry* may be a suppressor for inflammatory cytokine expression [16].

Many symptoms in allergic diseases exhibit prominent ~24-hour variation [18, 19]. For instance, in most allergic rhinitis patients, symptoms worsen overnight or early in the morning “morning attack” and often compromise night-time sleep, which results in poor daytime quality of life [20]. Such phenomena were actually recognized long before the birth of chronobiology. For example, Caelius (4th or 5th century AD) noted the frequent nocturnal occurrence of asthma attacks [21]. However, the precise mechanisms remain enigmatic.

Nakamura et al. have recently provided evidence that the circadian clock temporally controls IgE/mast cell-mediated allergic reaction* in vivo* [22]. They showed the time of day-dependent variation observed in IgE-mediated immediate type allergic reaction in the skin of wild-type mice (passive cutaneous anaphylactic [PCA] reaction) to be absent in* Per*2-mutant mice (mPer2^m/m^ mice) [23]. Most recently, they have also shown that the circadian clock present in mast cells primarily contributes to the generation of the daily rhythms observed in PCA reaction by temporally regulating Fc*ε*RI (the high affinity IgE receptor) expression and signaling [24]. Thus, allergic reaction also appears to be under tight control of the circadian clock. Future studies will ensure that many other chronic inflammatory diseases with circadian pathophysiology are under control of the circadian clock.

## 6. Conclusions

Recent strong molecular evidence shows an involvement of certain clock proteins in the circadian gating of immune responses. Importantly, new studies demonstrate direct interaction of certain clock proteins with regulatory pathways for cytokine expression in immune cells, which may contribute to shaping circadian immunity such as temporal variations observed in leukocyte trafficking, T cell development, innate immune defense against pathogens, and autoimmunity/allergy. Interestingly, disruption of the circadian clock appears to aggregate chronic inflammatory diseases such as RA via aberrant expression of inflammatory cytokines [6, 7, 17]. The findings may implicate that increased prevalence of human inflammatory diseases including autoimmunity and allergy in industrialized countries may be linked to modern life style chronically disrupting the circadian clock such as night shift work or jet lags. Thus, it will be a significant challenge in future how new basic studies on circadian control of immunity, especially on circadian regulation of cytokines, will be translated into prevention and treatment of infection, autoimmunity, and allergy.
